# Human Immunocompetent Model of Neuroendocrine Liver Metastases Recapitulates Patient-Specific Tumour Microenvironment

**DOI:** 10.3389/fendo.2022.909180

**Published:** 2022-07-13

**Authors:** Ewald Jan Doornebal, Nicola Harris, Antonio Riva, Ravi Jagatia, Michail Pizanias, Andreas Prachalias, Krishna Menon, Melissa Preziosi, Ane Zamalloa, Rosa Miquel, Yoh Zen, Michael Robert Orford, Simon Eaton, Nigel Heaton, John Ramage, Elena Palma, Rajaventhan Srirajaskanthan, Shilpa Chokshi

**Affiliations:** ^1^ Foundation for Liver Research, The Roger Williams Institute of Hepatology, London, United Kingdom; ^2^ King’s College London, Faculty of Life Sciences and Medicine, London, United Kingdom; ^3^ Institute of Liver Studies, King’s College Hospital and King’s College London, London, United Kingdom; ^4^ Liver Histopathology Laboratory, Institute of Liver Studies, King’s College Hospital, London, United Kingdom; ^5^ Great Ormond Street Institute of Child Health, University College London, London, United Kingdom; ^6^ Neuroendocrine Tumour Unit, ENETS Centre of Excellence, King’s College Hospital, London, United Kingdom

**Keywords:** tissue slices, neuroendocrine liver metastases, *ex vivo* model, soluble immunomodulators, immune checkpoint receptor, tumour modeling

## Abstract

Neuroendocrine liver metastases (LM-NEN) develop in a considerable proportion of patients with gastroenteropancreatic neuroendocrine neoplasms. There is a paucity of experimental models that accurately recapitulate this complex metastatic human liver microenvironment precluding scientific and clinical advancements. Here, we describe the development of a novel personalised immunocompetent precision cut tumour slice (PCTS) model for LM-NEN using resected human liver tissue. The histological assessment throughout the culture demonstrated that slices maintain viability for at least 7 days and retain the cellular heterogeneity of the original tumour. Essential clinical features, such as patient-specific histoarchitecture, tumour grade, neuroendocrine differentiation and metabolic capacity, are preserved in the slices. The PCTS also replicate the tumor-specific immunological profile as shown by the innate and adaptive immunity markers analysis. Furthermore, the study of soluble immune checkpoint receptors in the culture supernatants proves that these immunomodulators are actively produced by LM-NEN and suggests that this process is epithelium-dependent. This model can be employed to investigate these pathways and provides a powerful platform for mechanistic, immunological and pre-clinical studies.

## Introduction

Neuroendocrine neoplasms (NENs) are heterogeneous tumours that arise from cells of the neuroendocrine systems in almost any organ of the body. The incidence of NENs has had a 6-fold increase since 1973, reaching an annual age-adjusted incidence of 9 per 100,000 people in 2015 (1). The most common location for NENs is the gastroenteropancreatic (GEP) region and the liver constitutes a frequent metastatic site (liver metastases, LM) (2, 3). LM-NEN can lead to development of carcinoid syndrome and carcinoid heart disease. Regardless of the primary site, LM are a strong prognostic predictor of mortality, reducing considerably the overall survival compared to non-metastatic disease (4, 5). Although medical treatments can relieve symptoms and/or delay progression, the response rates are low and complete resection of LM-NEN tumours, which is rarely a curative option, is reserved for a minority (7-15%) of patients (6, 7).

Over the past decades, it has become increasingly clear that the pathobiology of tumours is far more complex than an accumulation of uncontrolled mitotic cells. Stromal cells, extracellular matrix, infiltrating immune cells, vasculature and other tissue-specific factors make up the tumour microenvironment (TME), a highly dynamic interactive network that has been shown to dictate tumour aggressiveness and drug resistance (8). Naturally, a change in the TME can result in a survival advantage for tumour cells that are most adapted to grow in that environment. This was recently demonstrated by Walter and colleagues, who found that small intestinal NENs have significant intertumoral heterogeneity between the primary and LM lesion, sometimes having a complete absence of common mutations (9). Additionally, studies have shown that up to 97% of LM-NEN are infiltrated with T-cells, immunosuppressive regulatory T-cells (T_regs_) and have surface expression of immune exhaustion markers (programmed cell death protein 1, PD-1 and programmed death ligand 1, PD-L1) (10, 11) making these tumours potential candidates for immune checkpoint inhibitor therapy, contrary to non-metastatic disease in which checkpoint inhibitors have had marginal success (12). It is therefore paramount to consider the relevant TME of the liver, in which LM-NEN have thrived, to investigate the nature of this cancer.

Besides the membrane-bound immune checkpoint receptors, there has been a growing interest in cell-free (soluble) forms of those immunomodulators (soluble immune checkpoint receptors (solCRs)), which are generated by alternative mRNA splicing or proteolytic ectodomain cleavage/shedding. Soluble CRs have been shown to maintain the functional properties that modulate the checkpoint receptor-mediated immune signalling (13, 14) and to be released in the liver-metastatic environment thereby modulating the intricate immunotolerant landscape of the liver, as it has been shown for other cancers with liver metastasis and in liver diseases (14, 15). An increasing number of publications have demonstrated that solCRs regulate anti-tumour immune response and there is intense research activity to elucidate the complex pathobiology of solCR, including which cell types produce solCRs, when and how (13). Amongst the most studied, sPD-1 has been reported to be able to interact both with PD-L1 and PD-L2 and to inhibit their interactions with the membrane-bound PD-1 as well as to activate CD8^+^ T cells (16, 17). In addition, prognosis has been shown to be considerably affected by the presence and concentration of solCRs in the circulation or the TME, their role as biomarkers has yielded promising results and has highlighted their use to monitor and predict response to therapy and disease progression in patients with various cancers (18, 19). Importantly, solCRs have been shown to have predictive value for the efficacy of checkpoint receptor therapies (20, 21). Finally, therapies targeting solCRs or clinical interventions aiming at their removal have been suggested as an adjunct strategy to immunotherapy and a potential avenue to tackle immunotherapy resistance (22). No studies to date have explored the therapeutic potential of solCRs as autologous biologicals or as biomarkers for immunotherapeutic strategies in LM-NEN, partially due to the paucity of experimental models that allow the investigation of solCRs and the human TME. This is an urgent unmet research need that significantly thwarts the advancement of our understanding of LM-NEN pathogenesis and the development of effective therapies (5, 23).

Various cell lines have been derived from NEN hepatic metastases such as GOT1, H-STS, CM and CNDT2 (24–26), but these cellular models present inherent shortcomings. For example, repeated cell passages favour highly proliferative adherent cell lines with chromosomal instabilities and loss of neuroendocrine features (such as chromogranin and achaete-scute homolog 1 expression), as such they bear little resemblance to the low-intermediate grade phenotype of well differentiated LM-NEN. Indeed, phenotypic and genetic investigations have evoked a debate regarding the authenticity and clinical value of these cell lines (27–29). Mouse models mimicking LM-NEN, such as the genetically engineered glucagon deficient GCGKO mouse model, simian virus 40 large tumour antigen transgenic mouse, or patient derived xenograft (PDX) models can overcome some of these challenges (30, 31), but lack in their ability to recapitulate the relevant (human) microenvironment, in which liver metastases thrive. Moreover, these models are not suitable for immunological studies or for testing immunotherapies, considering they are usually immunodeficient; even when immunocompetent, such as syngeneic mice, it would be difficult to predict how a mouse immune response translates to human immunity. Other experimental models relevant for the study of tumour biology of NENs include tumour organoids, spheroids and personalised cell cultures (32–36). Among the advantages, organoids can be derived from patient biopsies of primary tumours or metastases and in some cultures, they have shown to preserve the characteristics of the initial tumour and replicate *in vitro* the drug sensitivity of the patient of origin (32). However, a limiting drawback of NEN organoids is the low culture success rate (about 10%), which reduces the possibility of using this model for clinical applications and personalised medicine. Moreover, in organoids derived from very heterogenous tumours (common in NEN), a rapid increase in cells negative for the typical neuroendocrine markers synaptophysin or chromogranin A has been observed, suggesting a selective overgrowth of non-neuroendocrine cells underpinning the failure of these cultures to be consistently classified as NEN models (32).

In the present study, we describe the generation of a personalised *ex vivo* LM-NEN model using patient-derived precision cut tumour slices (PCTS). This organotypic culture of LM-NEN PCTS remains viable for at least 7 days (15 days for one patient) and preserves the original tumour proliferative capacity, differentiation, the native metastatic TME, stromal fraction and the distinctive/heterogeneous immune infiltrate. In addition, we demonstrate that this model can be used for immunological studies and to investigate the immunopathogenesis of LM-NEN. Finally, employing the PCTS model, we present the novel finding that solCRs are produced and released in the local TME by LM-NEN tumour slices.

## Materials and Methods

### Patient Recruitment and Sample Collection

This study was approved by the local Research Ethics Committee established by the Health Research Authority (REC reference 17/NE/0340; IRAS project ID 222302). Informed consent for the collection of plasma and surgical waste liver tissue was obtained from all patients in this study. We collected plasma from 28 patients and 17 healthy controls (**Table S1** for baseline characteristics) and tissue samples from 6 LM-NEN patients (**Table 1** for clinical characteristics). Patients positive for HIV or Hepatitis B or C were excluded.

**Table 1:** Clinical characteristics of patients recruited for PCTS generation.

Patient	Sex	Age	Ethnicity	Primary tumour	Pre-treatment	Tumour Grade	Ki67 (%)	Fibrosis Score	CgA
Pt#015	M	81	Caucasian	Lung	Hydroxycarbamide	G2	8	UA	+
Pt#045	M	58	Caucasian	Pancreatic	Ocreotide	G2	16.9	F0	+
Pt#051	F	51	Caucasian	Small bowel	–	G2	11	F0	+
Pt#062	F	52	Caucasian	Pancreatic	Streptozocin/Capecitabine	G1	3	F1-2	+
Pt#077	M	70	Caucasian	Small bowel	Lanreotide	G2	3.6	F0	+
Pt#106	F	69	Caucasian	Small bowel	Lanreotide/Ocreotide	G2	4.6	F0	+

CgA, Chromogranin A.

### Blood Plasma Isolation

Blood was collected in EDTA vacutainers (Becton, Dickinson and Company) and processed by centrifugation at 3,200 rcf for 10 minutes within 2 hours of obtaining the sample. Plasma was immediately stored at -80°C until analysis.

### Tissue Slice Preparation and Culture

**Figure 1:**
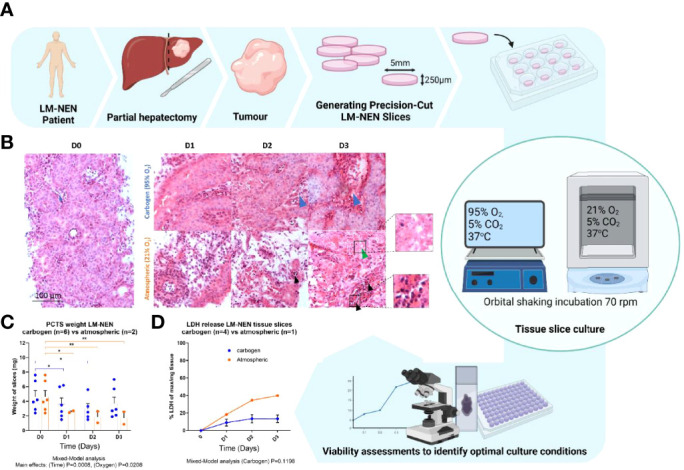
Precision cut tissue slices from LM-NEN require carbogen for tissue preservation beyond 3 days. **(A)** Schematic overview of experimental design. **(B)** Representative images of H&E staining of LM-NEN PCTS cultured in carbogen or atmospheric oxygen (n_patients_=3). Tumour stroma is highlighted by blue arrows. Areas of necrosis (green arrows) and apoptosis (black arrows) are magnified. **(C)** PCTS weight (in mg) was measured daily, each point represents a different patient and is the average of 3 technical replicates (slices), (n_patients_ indicated; *P ≤ 0.05, **P ≤ 0.01). **(D)** Normalised lactate dehydrogenase release (LDH) detected in PCTS culture supernatant. Each point is the mean ± SD of 3 supernatants per patient (n_patients_ indicated). Created with BioRender.com.

Human liver specimens were donated by patients who underwent partial hepatectomies for LM-NEN or primary liver cancer (**Table 1** and **Figure S6** for baseline characteristics). PCTS were obtained and cultured as previously described (37, 38). In short, the tissue specimens were flushed in the operating theatre with sterile ice-cold organ preservative solution (Belzer University of Wisconsin solution, UW) through open hepatic veins and arteries immediately after resection. Portions of tissue were cut from the samples and selected by liver histopathologists at a gross examination of the resection specimens, then transported to the laboratory, and processed within 3-4 hours. Tissue cores were cut from the tumour and surrounding tissue using a 5mm cylindrical hollow drill, and tissue slices with a thickness of 250 µm (corresponding to 10-15 cell layers) were cut using a Krumdieck tissue slicer (Alabama R&D). As slicing buffer, we used Krebs-Henseleit solution (2.5 mM CaCl_2_, 118 mM NaCl, 5 mM KCl, 1.1 mM MgSO_4_, 1.2 mM KH_2_PO_4_, 25 mM NaHCO_3_, 25 mM D-Glucose, 9 mM HEPES, all from Merck) pH 7.42 saturated with carbogen (95% O_2_/5% CO_2_). Each slice was then placed into a 12-well plate (Corning) and cultured with a recovery step of 2 hours in 1.5 mL supplemented Williams Medium E (sWME) (William’s E Medium (ThermoFisher Scientific), 5% Human AB serum (Pan-Biotech), Penicillin/Streptomycin (ThermoFisher Scientific), 2 mM Glutamine (ThermoFisher Scientific), ITS (10 mg/L Insulin + 5.5 mg/L Transferrin + 6.7 µg/L Sodium selenite, ThermoFisher Scientific), 1nM epidermal growth factor (ThermoFisher Scientific), 100nM Glucagon (Merck), 1µM Corticosterone (Merck), after which the medium was replaced, and the experiment started (Day 0). Culturing steps, including recovery, were performed under orbital shaking in a humidified incubator at 37°C in a sealed chamber saturated with carbogen (schematic in **Figure 1A**). Slices were cultured under these conditions for up to 15 days, with the media changed every 24 hours.

### Lactate Dehydrogenase

LDH release was quantified in the culture supernatants from tissue slices for the whole duration of the culture as a measurement of cell death. The supernatants were collected in triplicate for each timepoint, cleared from tissue debris by centrifugation at maximum speed (21,000 rcf) for 10 minutes, and frozen at -80°C for batch analysis. The colorimetric CyTox96 Cytoxicity Assay (Promega) was used to quantify LDH release, following the manufacturer’s instructions. As a positive control (total LDH content per mg tissue), tissue slices were collected in triplicate at the beginning of the culture (Day 0) and homogenized at 4°C using Precellys 1.4 mm ceramic bead tubes (Precellys CK14) containing sWME with 10% lysis buffer (provided with Promega LDH kit): 2 x 25 seconds on 5,500 rpm with a 30 second pause. The homogenate was then centrifuged at 21,000 rcf for 10 minutes to clear tissue debris and supernatants stored at -80°C until analysis.

### Cytokeratin 18

Full length (indicative of total cell death) cytokeratin 18 (CK18) and caspase-cleaved (indicative of apoptosis) cytokeratin 18 (cCK18) were measured in culture supernatants from tissue slices by M65 and M30 sandwich ELISA kits (PEviva) respectively according to manufacturer’s instructions. Results were plotted as a percentage of apoptotic cell death compared to total death.

### Adenosine Nucleotides

Adenosine and adenosine mono-/di-/tri-phosphate (AMP, ADP and ATP respectively) were quantified in liver slice homogenates by HPLC (JASCO Automated HPLC System) to assess intracellular ATP content (viability) and adenylate energy charge (metabolic capacity/state, equation 1). All steps were performed at 4°C up until sample loading, to prevent adenosine nucleotide degradation. In brief, tissue was collected in 500 µL ice-cold 1 M perchloric acid (VWR) and homogenised with 1 mm glass beads (Merck) using a Precellys homogenizer (Bertin Instruments) at 2 x 25 seconds on 5,500 rpm with a 120 second pause in-between on ice. The homogenate was stored at -80°C until analysis. Proteins were precipitated by adding 0.5 M Potassium Hydrogen Carbonate (VWR) until the pH was neutral. Protein precipitate was cleared by centrifugation at 13,000 rcf and the samples were then used for derivatization. Derivatization of the AMP, ADP and ATP molecules into fluorescent N6-etheno derivatives was performed by adding 100 µL of sample to 0.5 M Sodium Acetate pH 4.5 (VWR) and 0.2 M Chloroacetaldehyde (Merck) and reacting at 60°C for 40 minutes. The adenosine derivatives were then kept at 4°C. 20 ml of sample was analysed by reversed phase HPLC using a C18 column (Hypersil 5 ODS 4.6 × 150 mm, 3 µm, Sigma) at a flow rate of 0.8 mL/min for 40 minutes. The chromatography was achieved using a gradient from 100% mobile phase A (0.2 M Potassium Phosphate, pH 5.0, VWR) to 99% mobile phase B (0.2 M Potassium Phosphate, pH 5.0/10% Acetonitrile, Sigma). Detector excitation: 290 nm, emission: 415 nm, gain: 10x. Retention times (minutes) were ATP: 6.8 ± 0.1, ADP: 7.6 ± 0.1, AMP: 10.8 ± 0.2, Adenosine: 18.2 ± 0.4 and consistent for all samples and standards. Peak areas (AUC) of known standard solutions and samples were integrated using Unichrom v 5.0.19.1178 and used to calculate the concentration of the adenine nucleotides. Adenylate energy charge was calculated using the ATP, ADP and AMP concentrations with the following equation (39):


$$AEC=\frac{\left[ATP\right]+\frac{\left[ADP\right]}{2}}{\left[ATP\right]+\left[ADP\right]+\left[AMP\right]}$$

### Processing of Tissue Specimens, Histology and Immunohistochemistry

Tissue slices collected for histology or immunohistochemistry were placed in 10% neutral buffered formalin v/v and fixed overnight at 4°C. Fixed tissue slices were cryo-protected by placing the slices in 30% w/v sucrose (Merck) at 4°C until they sank to the bottom. As embedding matrix, 7.5% porcine gelatin (Merck) w/v + 15% sucrose w/v in PBS at 37°C was used. The blocks were then frozen in dry-ice cooled isopentane and stored at -80°C until sectioning.

For histological analysis, 10 µm sections were cut with the cryostat (Bright Instrument Co. Limited) and thawed in PBS at 37°C for 1 hour. Sections were stained for 1 minute with filtered Shandon’s Instant Haematoxylin (ThermoFisher Scientific). After dedifferentiation sections were stained with Eosin Y (Merck) for 10 seconds, dehydrated and mounted with DPX mounting medium (Merck). Images were taken at 200x magnification. For immunohistochemistry, 7 µm sections were cut and thawed as described above. The samples were permeabilized with 0.3% v/v Triton X-100 (Merck) in PBS for 5 minutes at room temperature. Excess permeabilization buffer was washed off with PBS and samples were blocked in blocking buffer (5% goat serum in PBS) for 1 hour at 37°C. Tissue sections were incubated with primary antibody diluted in blocking buffer (anti-Ki67 1:500, Novus Biologicals NB600-1209, RRID : AB_10001641; anti-Chromogranin A 1:500, NB120-15160, RRID : AB_789299; anti-CD3 Abcam, ab11089, CD3-12 RRID : AB_2889189) overnight at 4°C. The next day, slides were incubated for 1 hour at room temperature with secondary antibody, Goat-anti-Rabbit labelled with AlexaFluor 488 (Abcam, ab150077), AlexaFluor 555 (Abcam, ab150166) or AlexaFluor 568 (ThermoFisher, A-11004) diluted 1:300 in blocking buffer. Samples were then washed 4 times with PBS, mounted with Fluoroshield mounting medium with DAPI (Abcam, ab104135) and sealed with nail varnish. For the evaluation of apoptosis, tissue sections were stained using Dead-end Fluorometric TUNEL Kit (Promega, G3250). Images were made on a Cytation 5 imaging system (BioTek) at 10x magnification or with Olympus Fluorescence Microscope BX431 using UPlan FL N 20x and 40x objectives. Post-imaging analysis was performed in ImageJ. Any background reduction, brightness or contrast modifications were applied homogeneously across a complete image dataset.

Histopathological staining of liver specimens for diagnostic purposes was performed by Kings College Hospital Liver Histopathology Department. H&E staining was performed using standardised staining method for clinical histopathology. Immunohistochemical staining for chromogranin A and Ki67 was performed using the Leica-BOND-III automated staining platform. For chromogranin A, slides were pre-treated in citrate buffer for 20 minutes for heat-induced epitope retrieval, prior to staining with anti-chromogranin A primary IHC antibody clone 5H7 (Leica Biosystems, PA0515) at the commercially available concentration (>1.5 mg/L). For Ki67, slides were pre-treated in EDTA buffer for 20 minutes for heat-induced epitope retrieval, prior to staining with anti-Ki67 primary IHC antibody clone MM1 (Leica Biosystems) at the commercially available concentration (>1.9 mg/L). Interpretation of histology was confirmed by consultant histopathologists (R.M. and Y.Z.) at King’s College Hospital.

### Detection of Soluble Checkpoint Receptors by Luminex

The levels of solCRs in plasma and PCTS supernatants were analysed by multiplex Luminex technology using a commercially available custom 14-plex Immuno-Oncology Checkpoint Human ProcartaPlex Panel (ThermoFisher Scientific) according to the manufacturer’s instructions. The following 14 checkpoints were included in the panel: sBTLA; sCD28; sLAG3; sCD40; sCD80; sCD137; sCD152 (CTLA-4); sGITR; sHVEM; IDO-1; sPD-1; sPD-L1; sPD-L2; sTIM3. Measurements were performed using a Luminex MAGPIX Instrument and analysed using XPonent MAGPIX 4.2 and BioPlex Manager (Bio-rad, version 6). In samples where the solCR concentration was recorded as undetectable, a value corresponding to half the lowest detectable value was used for the purpose of analysis.

### PCR Array

Tissue slices were preserved in Allprotect Tissue Reagent (Qiagen) and stored at -80°C until processing. RNA, DNA and protein content were isolated with AllPrep DNA/RNA/Protein Mini Kit (Qiagen) according to manufacturer’s instructions. cDNA was synthesized with RT2 First Strand Kit (Qiagen) and analysed with Innate and Adaptive Immune Responses RT2 Profiler PCR Array (Qiagen) with RT2 SYBR^®^ Green qPCR Mastermix (Qiagen). qPCR array for Human Innate & Adaptive Immune Responses (Qiagen 330231 PAHS-052ZA, full list of genes available online) was run on ABI 7500 Real-Time PCR System with an initial denaturation step at 95°C for 10 minutes followed by 40 cycles of denaturation at 95°C for 15 seconds and annealing/extension at 60°C for 60 seconds.

### Image Analysis

Quantification of immunofluorescent and histological images (i.e. the number of Ki67, TUNEL positive nuclei or tumour epithelium percentage) was performed in ImageJ. For Ki67 and TUNEL, all cells in 3 images per sample were counted using a custom ImageJ macro that was created in ImageJ macro language. To quantify the content of tumour epithelium in the tissue slices, the H&E staining was used. The epithelium percentage was calculated by the following formula: Epithelium area/(Epithelium area + Stroma area) × 100%.

### Statistical Analysis

Continuous numerical variables were analysed by Mann-Whitney (MW) U rank-sum test (2 independent groups) or Kruskal-Wallis (KW) test (>2 independent groups) with Dunn’s *post-hoc* multiple comparison correction. Paired continuous numerical variables were compared by paired Wilcoxon Signed Rank test (2 groups) or (i) Repeated-Measures Two-way ANOVA with Holm-Sidak’s *post-hoc* multiple comparison correction or (ii) Mixed Model analysis with Tukey or Sidak correction for group/time-dependent cell-culture kinetics. Categorical variables were analysed by Chi-square test. Correlations were investigated by Pearson’s R or Spearman’s rho analysis as appropriate. The statistical analyses were performed with Prism GraphPad 8 and MS Excel 2016. Statistical significance was set at two-tailed alpha ≤ 0.05.

## Results

### PCTS can be Derived From Fresh Specimens of Neuroendocrine Liver Metastases and Successfully Cultured *Ex Vivo*

PCTS from LM-NEN were prepared from 5 resected human tumours and cultured at different oxygen concentrations for up to 3 days to identify the optimal conditions (**Figure 1** and **Table 1** for patient baseline characteristics). Based on our previous experience, tissue slices derived from tumour-free liver specimens necessitate high oxygen levels to maintain viability in culture (37). Considering the difference in metabolic requirements typical of cancer cells, both carbogen (95% O_2_/5% CO_2_) and atmospheric (21% O_2_) cultures for PCTS from LM-NEN were tested. In the carbogen environment, the tumour epithelium was observed at all timepoints and resembled the original tumour histology at baseline in terms of structure and diffuse morphology, according to independent histological evaluations conducted by expert LM-NEN histopathologists. Tumour stroma (**Figure 1B**, blue arrows) and epithelium were maintained. Tissue slices cultured at atmospheric oxygen levels lost tumour epithelial cells and tumour stroma over the duration of the culture period, with areas of necrosis (**Figure 1B**, green arrow) and apoptotic bodies (**Figure 1B**, black arrows) clearly visible at day 2 and day 3. The tissue slice integrity (as measured by the slice’s weight) was significantly affected by the duration of the culture period and the oxygen concentration (p=0.0008 and p=0.0208 respectively, Mixed-Model analysis) (**Figure 1C**). Multiple comparisons by time in culture and oxygen concentration found no statistically significant change in mass of the slices at day 3 compared to day 0 when they were kept in carbogen. In contrast, slices cultured in atmospheric oxygen lost significant weight compared to day 0 at all timepoints (p=0.0208). Cumulative lactate dehydrogenase (LDH) release further confirmed these findings as LM-NEN PCTS cultured in carbogen released less LDH over the duration of the culture period when compared to slices cultured in atmospheric oxygen (**Figure 1D**).

### PCTS From LM-NEN Tumours Maintain Viability, Metabolic Capacity and Histomorphology for Up to 15 Days

Based on the results shown in **Figure 1**, enhanced oxygenation (95% O_2_/5% CO_2_) was selected as the optimal condition for the culture of LM-NEN PCTS and, therefore, all the subsequent experiments have been performed in carbogen atmosphere.

**Figure 2:**
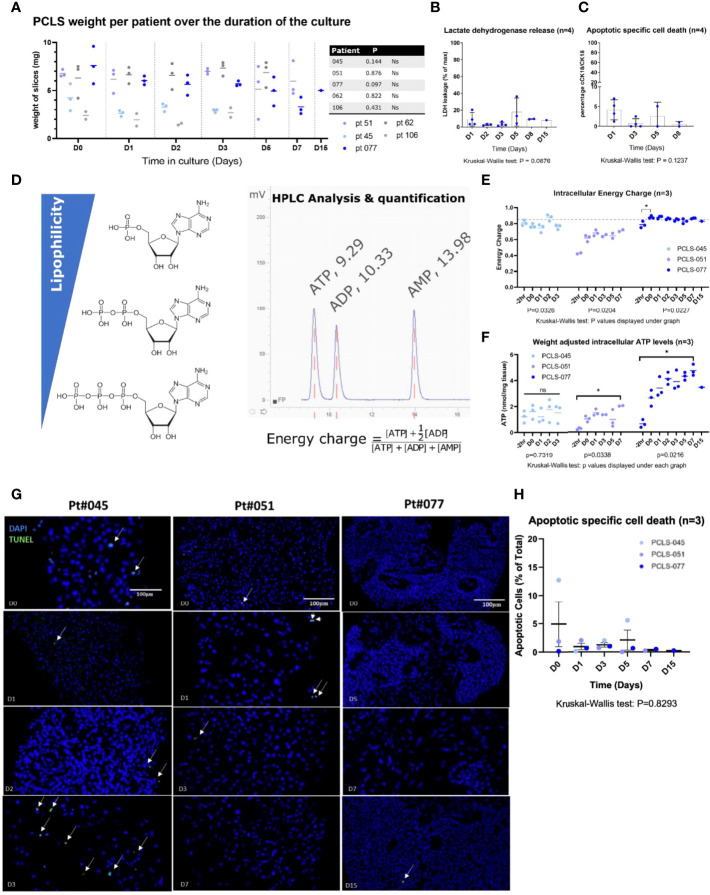
LM-NEN PCTS cultured in carbogen are viable for up to 15 days. **(A)** Tissue integrity was evaluated by measuring the slice weight over the duration of the culture period (at day 0, 1, 2, 3, 5, 7 & 15). Each point represents the weight of a single slice. 3 replicates per patient per timepoint are displayed as mean ± SD. Ns, P>0.05. **(B)** Weight-adjusted LDH leakage in PCTS supernatant throughout the culture was plotted as a percentage of the total LDH present per mg of tissue (determined separately for each patient). **(C)** Caspase-cleaved cytokeratin 18 (cCK18) indicative of apoptotic specific cell death is shown as a percentage of total cell death (full form cytokeratin 18, CK18). Datapoints in **(B, C)** represent individual patients and are an average of 3 replicates per patient. Bars indicate mean ± SD of 4 patients (D0-D8) or 1 patient (D15). **(D)** Overview of HPLC quantification of ATP, ADP and AMP and formula utilised to calculate the energy charge. Retention times for ATP, ADP and AMP (consistent across all runs) are indicated in the graph. **(E, F)** Intracellular energy charge and weight adjusted intracellular ATP levels in LM-NEN PCTS at indicated timepoints for patients with viable tumour epithelium (045, 051 and 077). Each datapoint represents a single slice (*P ≤ 0.05). **(G)** Representative images showing apoptotic nuclei stained by TUNEL (green, indicated by white arrows) in tissue slices generated from 3 patients; 3 slices (D0-D7) or 1 slice (D15) were analysed per patient at each indicated timepoint. **(H)** TUNEL positive nuclei were quanitfied ans presented as %Apoptotic cells of total number of cells per image. Data are shown as mean ± SEM of 3 patients and each dot represents a single patient.

Long term viability was assessed by measuring slice integrity (weight), LDH release, apoptotic cell death and intracellular ATP levels up to 15 days (**Figure 2**). Tissue slice weight remained constant over the duration of the culture period for all patients (**Figure 2A**). Release of LDH was always lower than 20% of the total expected LDH in the tissue slices, and apoptotic cell death was consistently lower than 5% evaluated both on supernatants (cCK18) and on the tissue *via* TUNEL assay (**Figures 2B, C, G, H**). In slices from patients 051 and 077, the intracellular ATP content significantly increased after the 2-hour recovery period and stabilised at 1.38 and 3.86 nmoles/mg tissue, respectively (**Figure 2E**). The ATP content for slices from patient 045 remained stable at 1.53 nmoles/mg tissue throughout the culture period. The adenylate energy charge (AEC) showed a similar trend to the ATP levels (**Figure 2F**). The AEC for slices from patients 045, 051 and 077 was 0.81, 0.43 and 0.79 before the recovery and stabilised at 0.80, 0.66 and 0.85 for the remainder of the culture period, respectively. The consistently low levels of LDH and apoptotic cell death markers combined with the stabilisation of the intracellular ATP and AEC levels after the recovery step indicate that the cells in the tissue slices maintained a good viability for the duration of the culture period.

**Figure 3:**
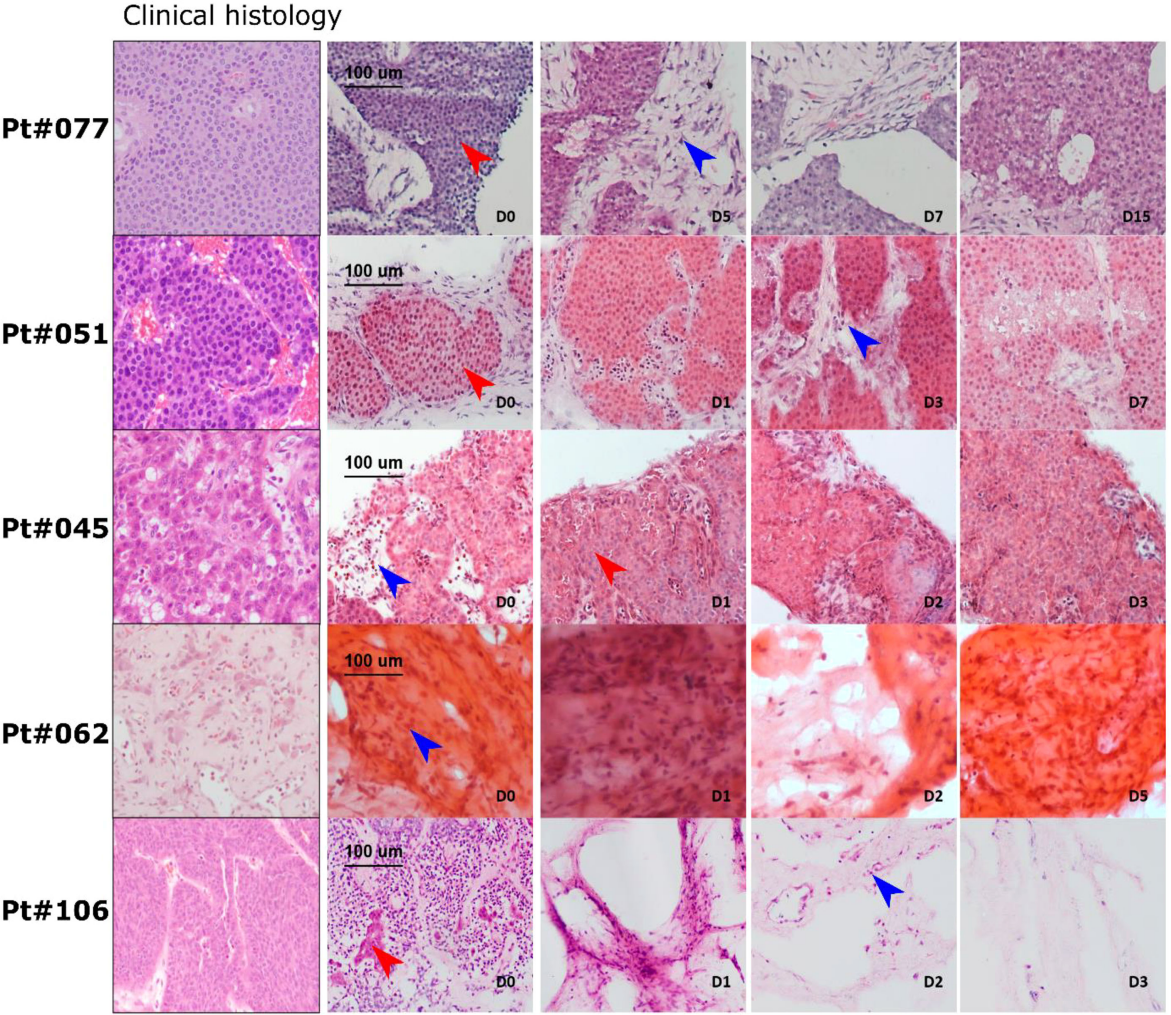
LM-NEN PCTS retain patient specific histoarchitecture in culture. Representative H&E images of tissue slices generated from 5 patients; 3 slices (D0-D7) or 1 slice (D15) were analysed per patient at each indicated timepoint. Tumour epithelium is indicated by red arrows and tumour stroma by blue arrows. Histoarchitecture was compared with clinical histopathology staining (left column) for each patient.

**Figure 4:**
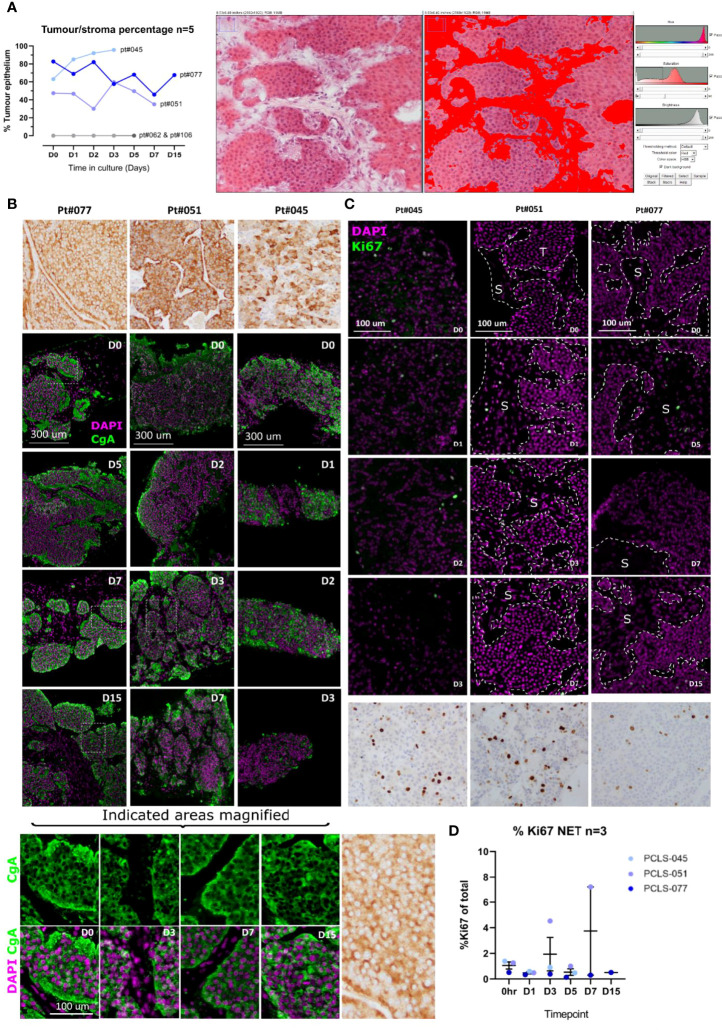
LM-NEN PCTS retain key molecular features associated with tumour grade and neuroendocrine differentiation. **(A)** The areas of tumour epithelium and stroma (highlighted in red) were measured in the H&E images of each patient throughout the culture using ImageJ as indicated on the right panel (n_patients_=5) and the epithelium percentage is shown for each patient per timepoint. Patients 062 & 106 lacked tumour epithelium hence % = 0. **(B)** Chromogranin A (CgA) staining in brown or green and DAPI (purple) on epithelial LM-NEN tissue slices at indicated timepoints. **(C)** Representative images for Ki67 staining (positive nuclei in green or brown) and DAPI (in purple) at indicated timepoints. Areas of tumour and stroma are indicated with T and S, respectively. Images in **(B, C)** at D0-D7 are representative of 3 technical replicates per patient, for D15, 1 replicate. **(D)** Ki67 was quantified in 3 images per patient and presented as %Ki67 of total number of cells per image. Data are shown as mean ± SD of 3 patients and each dot represents a single patient.

Histological and tissue structure analysis of the PCTS was performed and cross-matched with the routine clinical histopathology H&E staining (**Figure 3**). For all patients except patient 106, the PCTS retained near-intact histomorphology when directly compared to the clinical histopathology reference staining at all timepoints. Tissue from patient 106 did contain viable tumour, but after day 0 the tissue slices did not show any viable tumour epithelium and were therefore classed as stromal slices. Tissue from patient 062 did not have viable tumour epithelium at the start of the culture and consisted of stroma. Although these tissue slices comprised mainly tumour stroma, the non-epithelial stromal cells remained detectable and viable in the tissue slices over the duration of the culture period (**Figure 3**, blue arrows). Viable tumour epithelial cells could be seen in all tissue slices for patients 045, 051 and 077 (red arrows). The tissue structure in slices from patient 045 resembled typical pancreatic NEN with mild nuclear atypia, round and oval in a trabecular growth pattern (ribbon like appearance and gland like formations). Slices from patients 051 and 077 displayed characteristic trabecular, insular growth and uniform, small and round nuclei with granular chromatin patterns often referred to as ‘‘salt and pepper’’ morphology which is typical for small bowel NEN. Fibrotic stroma surrounding the tumour epithelial nests could be clearly recognised. Again, stromal cells and immune cells were visible throughout the tumour stroma and the initial tumour epithelium percentage was maintained constant for the duration of the culture period for all patients (**Figure 4A**).

### Neuroendocrine Differentiation Markers Are Maintained in LM-NEN PCTS

The neuroendocrine differentiation of the tumour cells within the tissue slices was assessed. All the LM-NEN samples utilised to obtain PCTS expressed chromogranin A (CgA), as confirmed by the CgA staining performed by the clinical histopathologist (**Figure 4B**, top row). In the PCTS cultures, CgA expression was observed for all patients and remained stable over the culture period. The CgA staining co-localised with the tumour epithelium, not with the stroma, and was matched with the clinical histopathology reference staining for each patient. As expected, the CgA staining could be detected within the cytoplasm and not the nucleus of the tumour cells (**Figure 4B**, magnified panel).

### Proliferative Capacity Remains Stable in LM-NEN PCTS

The proliferative capacity was assessed to investigate if our culture settings were altering the inherent proliferation grade of the tumour. In accordance with the guidelines from the World Health Organization 2019 Classification of Tumours of the Digestive System (40) both the number of Ki67 positive cells (%Ki67) and the mitotic rate were quantified. Given the limited size of PCTS compared to the clinical specimens analysed at histopathology, the %Ki67 was determined based on all the tumour cells in the examined section, and not in selected areas with concentrated mitosis (proliferation hotspots), as is done by routine histological assessment clinically. The level of %Ki67 for the tissue slices at the start of the culture (day 0) was therefore lower than the reported levels from the clinical histopathologist (**Figures 4C, D**). In the LM-NEN PCTS at day 0, the levels of Ki67 positive cells (for patients 045, 051 and 077, respectively) were 1.39, 1.27 and 0.51% compared to 16.9, 11.0 and 3.6% as reported by the clinical histopathologist. However, representative images from histopathology for the corresponding patients illustrating areas outside the hotspots confirm the low levels of proliferation, reflecting rates observed in the PCTS (**Figure S1**). Importantly, the proliferative capacity in the slices did not significantly change over the duration of the culture (p=0.2871) and remained constant at 2.90 ± 2.88, 0.37 ± 0.14 and 0.84 ± 0.41 for patients 045, 051 and 077, respectively. In addition, the rate of mitosis in the LM-NEN PCTS was evaluated. All the samples included in the study were well-differentiated tumours with rare mitotic figures, therefore the number of mitotic cells in the PCTS was extremely low and close to zero for most patients (data not shown, example of one mitotic nucleus detected in patient 051 **Figure S2**).

### LM-NEN Slices Are Immunocompetent and Produce Soluble Forms of Checkpoint Receptors

**Figure 5:**
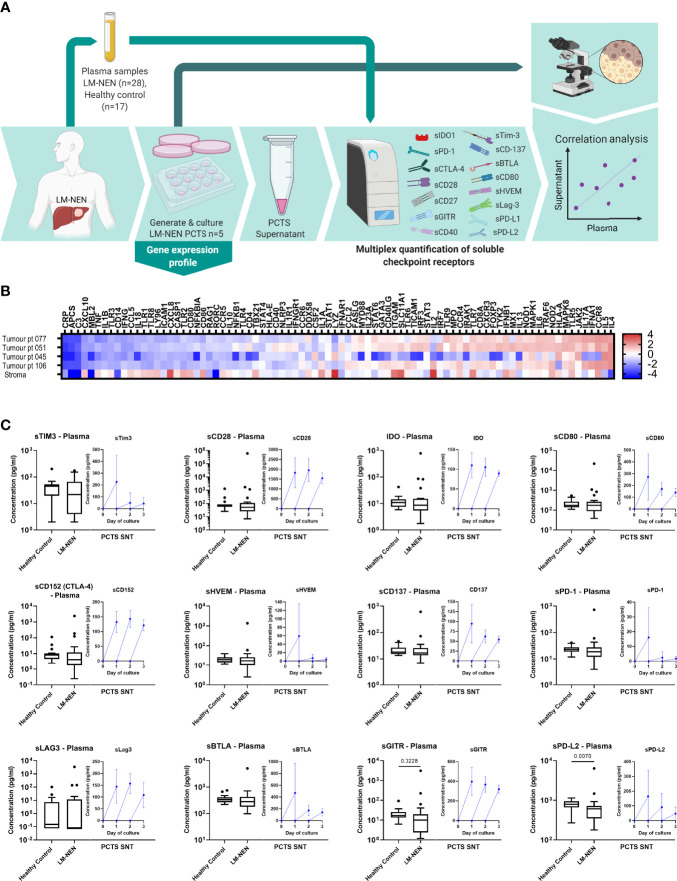
LM-NEN PCTS retain innate and adaptive immunity markers and release soluble checkpoint receptors (solCRs) in the supernatant. **(A)** Schematic representation of the experimental design. Plasma samples were collected from LM-NEN patients (n=28) and healthy controls (n=17). Additionally, PCTS were generated from patients with LM-NEN (n=5) and culture supernatants collected at day 1, 2 and 3. solCRs were quantified in plasma and supernatants using Luminex. In parallel, gene expression analysis of innate and adaptive immunity markers was performed at day 1. Created with BioRender.com. **(B)** Heatmap showing the differential gene expression of immune cell markers in tumour versus surrounding tumour-free liver tissue in slices derived from patients with LM-NEN (n=4) and primary liver cancer with a prevalence of stroma, stroma (n=1). **(C)** Levels of solCRs in LM-NEN plasma (black box and whiskers plots) and slice supernatants at day 1, 2 and 3 (blue line graphs, mean ± SD of 3 replicates per patient). The supernatant was refreshed daily hence the levels are always displayed from y=0.

To assess the immunocompetency of LM-NEN PCTS, 84 markers for innate and adaptive immune cells were investigated for all patients with viable tumour epithelium and one patient that contained no viable tumour epithelium (Patient 106) (**Figures 5A, B**). Each patient had differences in their immunological profiles and for patient 045 we found low expression levels of most genes, characteristic of a ‘‘cold’’, non-infiltrated TME. However, IL-4 and IL-5 levels were consistently increased in tumour tissue slices compared to tumour-free slices from patient-matched surrounding tissue, with an average fold change of 24.80 and 15.43, respectively. In addition, immune signatures typical of T_reg_ such as FOXP3, CCR4 and CCR8 were increased in LM-NEN PCTS, while expression of co-stimulatory molecules CD80 and CD86 were decreased. Interestingly, patient 106 had a similar expression pattern compared to patient 077 and 051, suggesting that the immune cell infiltrate was not affected by the lack/presence of tumour epithelium. Additionally, we measured the same factors in tissue slices with a prevalence of stroma vs. epithelium but derived from a patient with a primary liver tumour and this analysis showed a completely different immunological landscape (**Figure 5B**). The presence of T cells (CD3^+^) on PCTS derived from patients 051, 045 and 106 was also confirmed by IF at day 1 and day 7 (**Figure S3**).

To further investigate the immunological characteristics of the tumour slice culture, the levels of soluble (s)BTLA, sCD28, sLAG3, sCD40, sCD80, sCD137, sCD152 (CTLA-4), sGITR, sHVEM, IDO-1, sPD-1, sPD-L1, sPD-L2 and sTIM3 were measured in PCTS supernatants of patients with LM-NEN. We observed that tissue slices consistently released solCRs in the tissue culture media after each day in culture, and for most of the molecules, the detection of solCRs was constant for the entire culture duration (**Figure 5C**, blue line graphs).

In addition, we quantified the same 14 solCRs analysed in the slice supernatants in plasma samples collected from 28 LM-NEN patients and 17 healthy controls (**Figures 5A, C**, black box and whisker plots). For most of the molecules, no difference was observed between the LM-NEN patients and healthy controls, besides sGITR and sPD-L2 which were lower in patients (P=0.028 and P=0.007, respectively). Interestingly, in patients with pancreatic LM-NEN, plasma solCRs were consistently lower for all analytes when compared to gastrointestinal LM-NEN (**Figure S4**). In addition, for some of the patients (n=5) we could correlate the plasma solCRs with the levels in PCTS culture supernatants, but only a weak correlation was found (**Figure S5**). Overall, these data suggest that while there is no aberrant systemic regulation of solCRs in LM-NEN, there seems to be a consistent local production of solCR in the LM-NEN TME.

**Figure 6:**
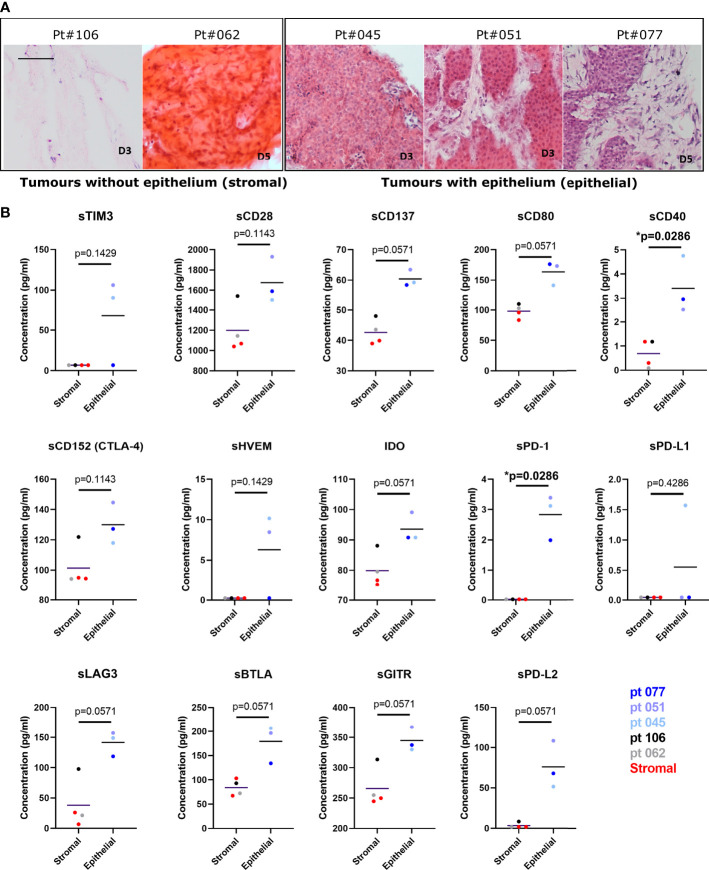
The release of soluble checkpoint receptors (solCRs) by LM-NEN PCTS is dependent on the presence of tumour epithelium. **(A)** Histological assessment of the content of stroma and epithelium in LM-NEN PCTS at day 3 or 5 in culture (see **Figure S3** for images related to HCC with prevalent stroma). **(B)** solCRs quantified in supernatants at day 3 in culture derived from LM-NEN slices with prevalent epithelium (n_patients_=3) and slices with prevalent stroma (n_patients_=4, 2 LM-NEN + 2 primary liver cancer). Each datapoint indicates the average of 3 technical replicates (slice supernatants) per patient. LM-NEN in blue and grey, stroma primary liver cancer in red. P values following the comparison of stroma slices vs epithelial LM-NEN slices are indicated in graphs.

Further analysis of the solCR data was performed after stratifying the patients based on the histological features of their tissue slices. A consistent difference was observed in solCRs between LM-NEN PCTS containing viable tumour epithelium (patients 045, 051 and 071) compared to PCTS that consisted of only stroma (patients 062 and 106). The concentration of all the analysed solCRs was elevated in the culture medium of the epithelial LM-NEN PCTS compared to supernatants from stromal slices (**Figure 6**). In the latter, all the checkpoints were low but within detectable range besides sHVEM, sTIM3, sPD-1, sPD-L1 and sPD-L2. We proceeded by interrogating the solCR profile of tissue slices generated from other liver tumours (hepatocellular carcinoma and cholangiocarcinoma) with a prevalence of stroma (**Figure S6**). We found similar levels (low) of solCR production as from the stromal LM-NEN slices. After combining the data derived from all the stromal slices (LM-NEN and HCC), we observed that the release of sCD40 and sPD-1 was significantly higher in the LM-NEN epithelial tumour slices (p=0.0286) and a similar trend was detected for sCD137, sCD80, IDO-1, sLAG3, sBTLA, sGITR and sPD-L2 (p=0.0571). In contrast, sCD28 and sCD152 were similarly expressed in epithelial and stromal slices (p=0.1143). These findings suggest that solCRs are produced by the LM-NEN epithelial cells – or in response to the presence of LM-NEN epithelial cells – and not by the hepatic TME in the absence of tumour cells, or that a decrease/change in the stromal component can affect the production of solCRs.

## Discussion

Modern therapeutic strategies for cancer act beyond direct cytolysis of tumour epithelial cells and often target different tumor components, such as the TME and the immune infiltrate. This change of focus in the study of tumour biology, combined with the rise of precision medicine, has revealed a lack of appropriate and more complex models for pre-clinical studies. The development of the tissue slice *ex vivo* platform described here can significantly advance our understanding of LM-NEN pathobiology and potentially accelerate drug development in a research area that lacks basic and translational tools and studies. Human tissue slice models have been validated before to mimic breast, pancreatic, and metastatic colorectal liver tumours *ex vivo* (41–43). Our approach directly addresses the main limitations of the existing LM-NEN pre-clinical disease models, namely lack of TME or immune compartment and maintenance of tumour-specific characteristics. The precision cut LM-NEN slices retain tumour grade, neuroendocrine differentiation, the resident immune infiltrate and tumour stroma interactions for up to 15 days *ex vivo*, which exceeds the viable lifespan reported by other tissue slice studies and is on par with a recently reported three-dimensional (3D) primary cell culture approach for neuroendocrine tumours (33). The LM-NEN slice model focuses on the clinically relevant subgroup of neuroendocrine liver metastases. The slicing technology offers several advantages inherent to the technique, allowing the recapitulation of the original tumour with near *in vivo*-like accuracy in a controlled and highly reproducible laboratory setting. One of these advantages is the ability to reproduce the native liver-specific microenvironment in which these metastases flourish, including the stromal compartment and the tissue-resident immune landscape. LM-NEN slices add to the organotypic model recently developed by Herring et al. for primary neuroendocrine tumours, based on the 3D culture of patient-derived tissue fragments in a flow-perfusion bioreactor (36). However, the two models are substantially different in the amount of starting material required. In the system described by Herring et al., the minimum amount of tissue necessary for the perfusion channels of each bioreactor to work is 250mg (36). This quantity constitutes a single biological replicate (or test condition) and therefore, the analysis of different treatments would necessitate much more tissue, which is rarely available in NEN. In the PCTS model, approximately 50 slices were obtained from a similar amount of tissue, allowing for expanded analysis and sufficient replicates for consistent multi-parameter and/or longitudinal investigations.

Recurrence following curative resection of LM-NEN is common (>50% cases), and this is when tissue is often available for research, affording the possibility to use this model as a proactive drug screening platform, as shown for other tumour slices (42). Drug testing on PCTS would allow the selection of patient/tumour-specific efficacious therapeutics *ex vivo* to prevent or treat recurrent hepatic disease (3, 44). That said, a limitation of this study is that only specimens from patients with grade 1 and 2 of well differentiated neuroendocrine tumours were utilised to produce tumour slices. Grade 3 or poorly differentiated neoplasms, associated with the poorest prognosis, were not represented as only a selected number of these patients are suitable for surgical resection of liver metastases. This issue can be overcome by deriving and culturing tumour fragments from liver biopsies, which can be obtained across all tumour grades. However, preliminary experiments performed in our laboratory suggest that technical limitations, including specimen size and tissue fragmentation due to the biopsy procedure, severely impact the feasibility, success rate, and viability of the tissue culture with fragments (data not shown). Notably, most patients in the current study were treated with somatostatin analogues, but this did not affect the viability of the tumour tissue *ex vivo.*

Despite the extraordinary success of immuno-oncology in several cancer types, there is still reduced or inconsistent evidence of the effects of immunotherapy on NENs (45). This is principally explained by the limited knowledge of the neuroendocrine immunological landscape and the lack of appropriate models to perform molecular studies. We demonstrate that LM-NEN PCTS maintain proliferative and metabolic capacity and replicate distinct immunological phenotypes such as ‘‘hot’’ and ‘‘cold’’ tumours, typical of the original tissue. For example, markers of immunosuppressive T_reg_ were upregulated, suggesting that LM-NEN PCTS could capture the infiltrated but suppressed immunity associated with immune exhaustion, although this would need to be confirmed as T_reg_s are not the only cells in the TME that can express FOXP3. The main scope of the current article is to report the development and characterisation of an immunocompetent organotypic model for the investigation of LM-NEN, which will fill the gap between pre-clinical and clinical research in this field.

Additionally, this study has revealed for the first time that LM-NEN slices in culture produce and release detectable levels of solCRs and this further validates the PCTS model for studies aimed at investigating the novel role of solCRs in neuroendocrine liver metastases. Despite the importance of solCRs in affecting the homeostasis between tumour and immune response (13), no investigations to date have described the influence of these soluble forms in the context of LM-NEN. The quantification of solCRs in the systemic circulation (plasma) of LM-NEN patients did not show significant differences compared to healthy controls, but the analysis of PCTS supernatants demonstrated that solCRs are strongly and consistently produced locally by LM-NEN and this occurs in an epithelium-dependent manner. For example, sPD-1 was significantly increased in the supernatant of epithelial LM-NEN tissue slices compared to stromal slices, whilst sPD-L1 did not show the same trend. This is relevant because although sPD-1 may be able to exert anti-immunosuppressive effects, it might also act as a decoy for therapeutic antibodies directed against membrane bound PD-1, such as Nivolumab, thereby negatively affecting the patient response to immunotherapy and outcome (46). In addition, the release of solCRs by LM-NEN seems to be dependent on the presence of tumour epithelial cells and not solely reliant on the TME or presence of immune infiltrate. In fact, the lack of viable tumour epithelium in the stromal PCTS (patients 062 and 106) was not associated with diverse levels of immune infiltrate, as indicated by the PCR panels and T cell detection by IF. Moreover, viable non-epithelial cells were identified by histology both in stromal and epithelial slices. PCTS from patient 045, whilst containing mainly tumour epithelium, had a distinctively ‘cold’ microenvironment, but solCRs in the supernatant were comparable to the levels detected in the cultures from other epithelial PCTS (patients 051, 077). Whether the solCRs are produced by LM-NEN tumour epithelium or by other cells in response to the presence of tumour epithelium is currently unknown and requires further investigation.

Overall, small sample size notwithstanding, our results suggest that production of solCRs involves tumour cells and possibly immune resident and/or non-parenchymal/stromal cells. It is difficult to accurately pinpoint which cell subsets actively produce these paracrine immune factors, but in the slice model, PCR array characterisation suggests immune involvement. In the solCR analysis, only the levels of two molecules (sCD40 and sPD-1) were significantly increased in epithelial compared to stromal PCTS culture supernatants. However, it may be relevant to highlight that a clear trend was observable in all the other 12 solCRs and increasing sample sizes may have revealed significant differences.

Importantly, different levels of solCRs and a diverse intra-tumoral immune compartment were observed among the patients, highlighting the utility of PCTS for precision medicine and as a tool to identify subjects that may respond to current and emerging therapies. Indeed, studies investigating immune checkpoint inhibitors in neuroendocrine malignancies have indicated that only a small subgroup of patients may benefit from treatment (47–49) and the technology presented here could aid the identification of these patients.

In summary, this study demonstrates the value of PCTS technology for investigating the (immuno)pathobiology of neuroendocrine liver metastasis and developing complex personalised disease models of LM-NEN, especially for studies focusing on TME and stroma-epithelium interactions. Furthermore, as reported for other solid tumours, LM-NEN PCTS could constitute an excellent platform for drug screening and studies exploring the increasingly more common group of therapeutic strategies that do not target the replicating tumour epithelial cells, but other components, such as immunotherapies. Finally, although PCTS require specific equipment, tissue source and expertise, the cost-effectiveness, efficiency and speed of preparing human LM-NEN PCTS from surgically resected tissue for discard make this model globally applicable.

## Data Availability Statement

The PCR Array data discussed in this publication have been deposited in NCBI's Gene Expression Omnibus accession number GSE206828 (Doornebal et al., 2022). Further inquiries can be directed to the corresponding authors.

## Ethics Statement

The studies involving human participants were reviewed and approved by Research Ethics Committee established by the Health Research Authority (REC reference 17/NE/0340; IRAS project ID 222302). The patients/participants provided their written informed consent to participate in this study.

## Author Contributions

ED: experimental work, data analysis, interpretation, manuscript writing and preparation. NHa, RJ: experimental work, data analysis. AR: data analysis, interpretation, manuscript review. AZ, MPr, MPi, AP, KM & NHe: patient recruitment/consent and sample collection. RM & YZ: interpretation of histology. SE, MO: data interpretation, manuscript review. EP & SC: experimental design/supervision, data analysis, interpretation, manuscript writing and preparation. RS & SC: study conception/design and manuscript review. All authors contributed to the article and approved the submitted version.

## Funding

The work described in this manuscript was funded by the Foundation for Liver Research (FLR) and the Neuroendocrine Research Foundation (NETRF, Pilot project award).

## Conflict of Interest

The authors declare that the research was conducted in the absence of any commercial or financial relationships that could be construed as a potential conflict of interest.

## Publisher’s Note

All claims expressed in this article are solely those of the authors and do not necessarily represent those of their affiliated organizations, or those of the publisher, the editors and the reviewers. Any product that may be evaluated in this article, or claim that may be made by its manufacturer, is not guaranteed or endorsed by the publisher.
