# Supplementary material for: Human Immunocompetent Model of Neuroendocrine Liver Metastases Recapitulates Patient-Specific Tumour Microenvironment
Source: Front Endocrinol (Lausanne). 2022 Jul 13;13:909180. doi: 10.3389/fendo.2022.909180 (PMC9326114; doi:10.3389/fendo.2022.909180)
Supplement: Supplementary file 1 [file DataSheet_1.docx]

**
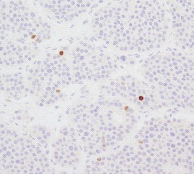

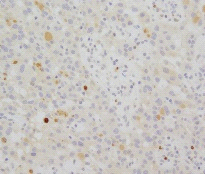
** **Pt#045 Pt#051 Pt#077**

**Figure S1.** Representative images for Ki67 staining (positive nuclei in brown, magnification x200) from clinical histopathology for the indicated patients. The areas imaged are outside the proliferative hotspots and show a very low number of positive nuclei.


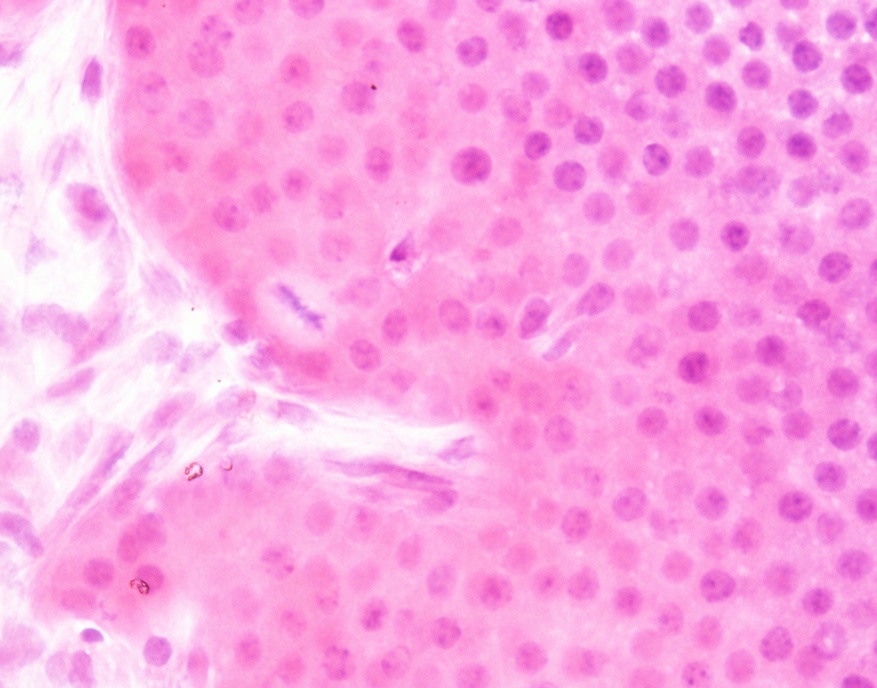


**Figure S2.** Representative image of a mitotic nucleus in PCTS from patient 51 (arrow, magnification x40).


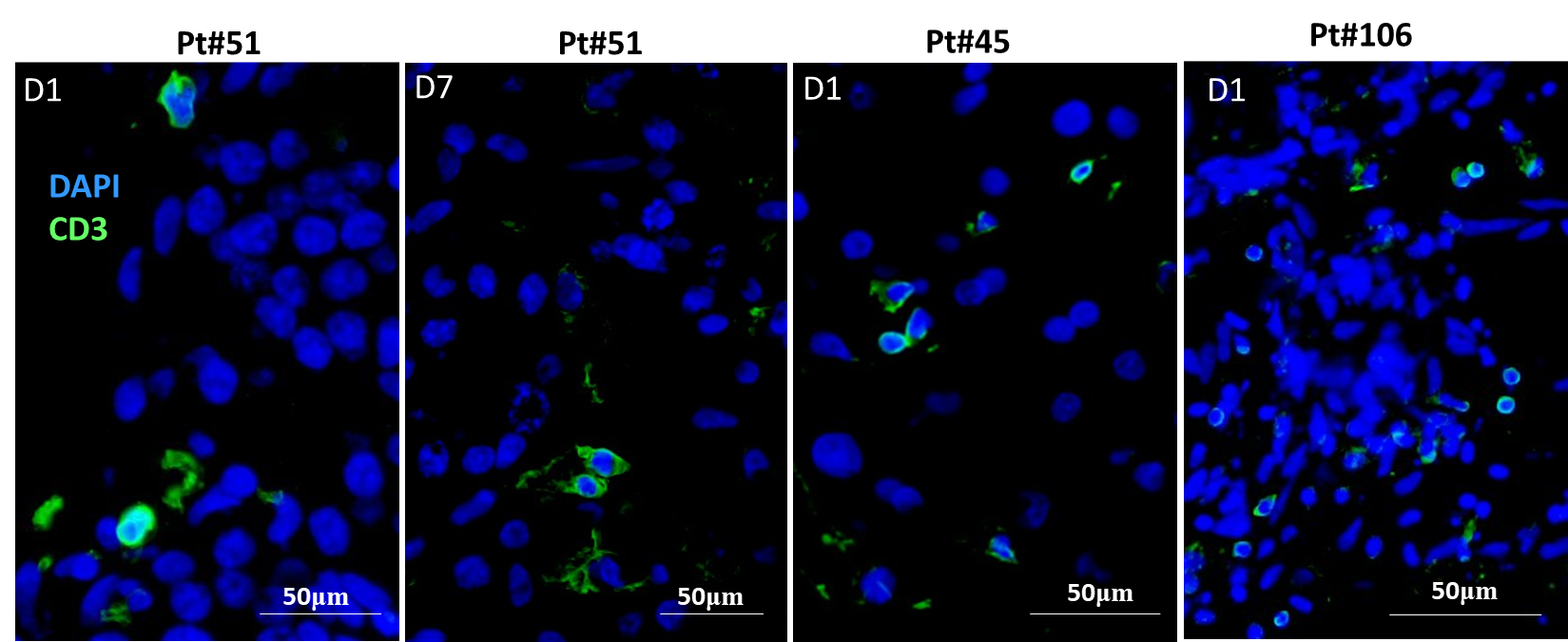


**Figure S3.** Representative image of CD3 positive cells (green) in PCTS from the indicated patient after 1 or 7 days in culture.


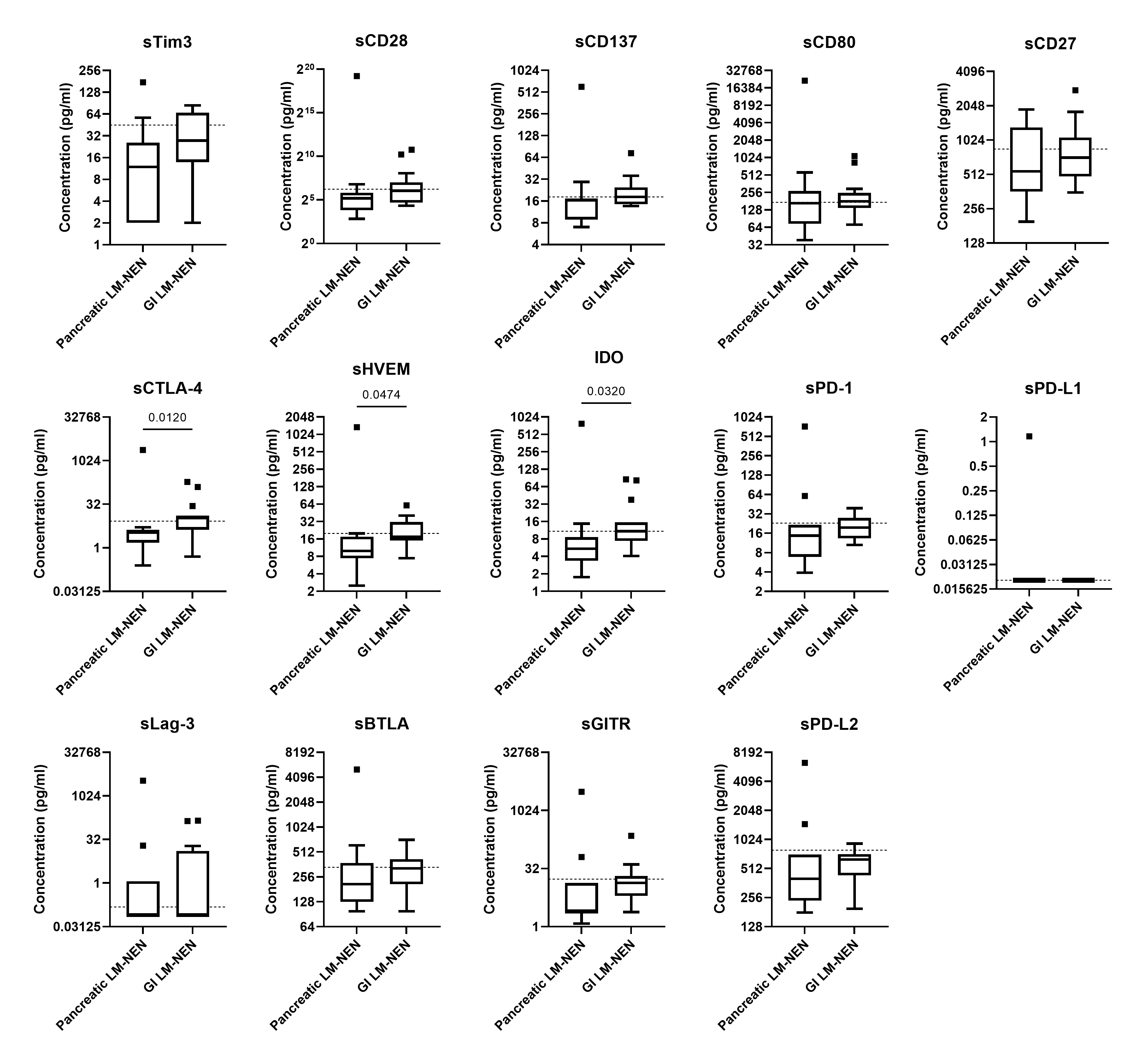


**Figure S4.** Soluble checkpoint receptors measured in plasma samples from patients with pancreatic (n=11) and gastrointestinal LM-NEN (n=15). Dotted lines indicate median sCR concentration in healthy controls.


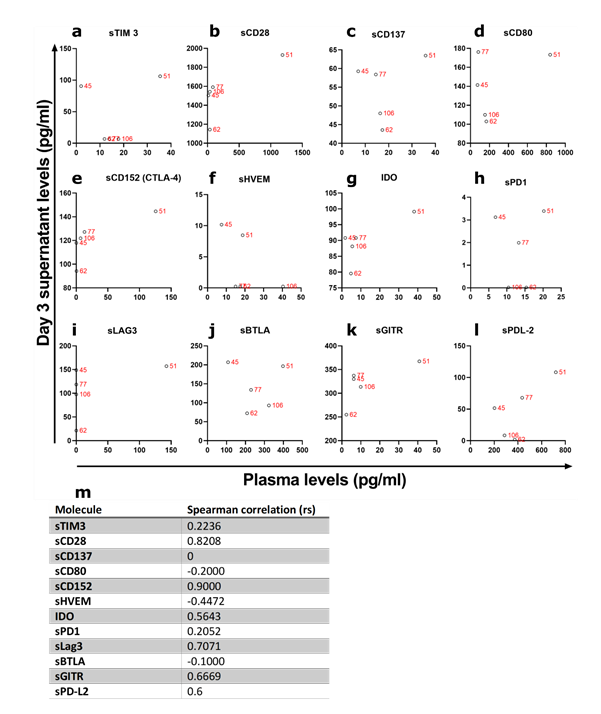


**Figure S5.** Soluble checkpoint receptor (solCRs) levels in LM-NEN plasma and matched tissue slice supernatants shows low levels of correlation. **a-l**, X-Y plots for the individual analytes for 5 matched plasma and supernatant samples. Each point represents the plasma level (X-axis) and the average of 3 replicates of day 3 supernatant levels (Y-axis) of the indicated solCR. Patient identification numbers are shown in red next to the data point. **m,** Table with spearman correlation (rs) for the indicated solCR.


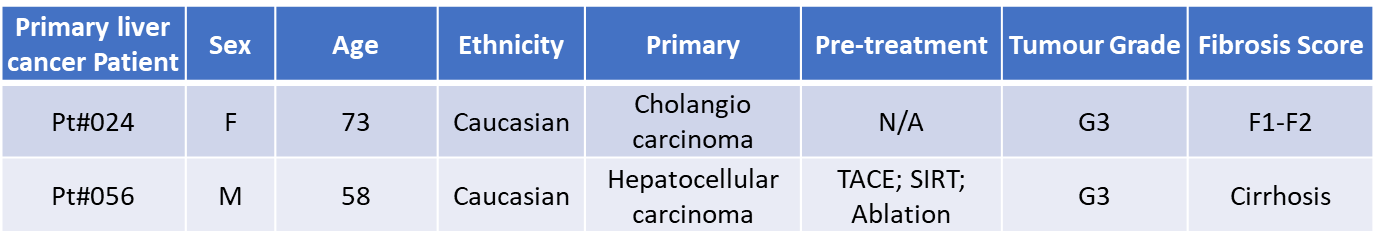


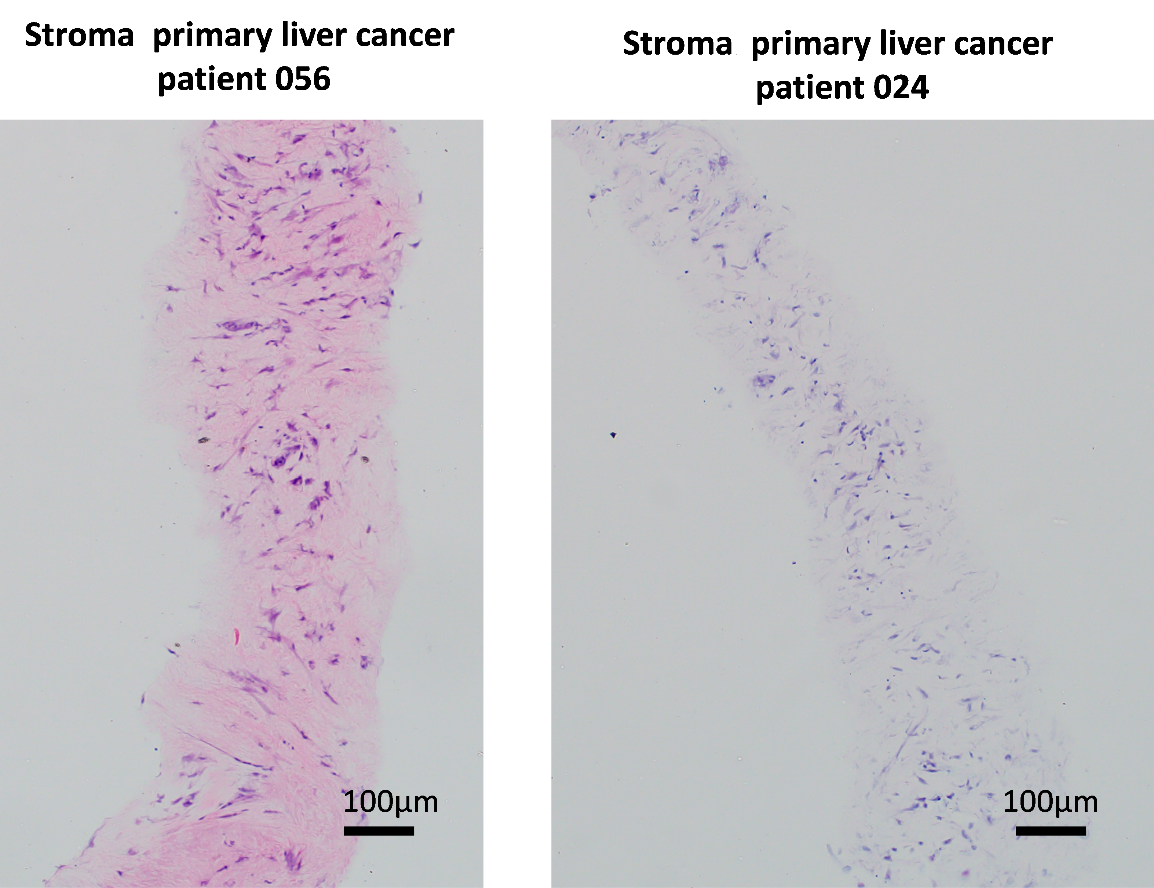


**Figure S6.** Clinical characteristics of the patients with primary liver cancer and representative pictures of tumour slices stained with H&E. The histological analysis of the tumour slices derived from these patients shown a prevalence of stroma vs. epithelium.

|  | | **NET** | | **Ctrl** | |
| --- | --- | --- | --- | --- | --- |
|  |  |  | % |  | % |
| **n** | | **28** | | **17** | |
| **Sex** | **M** | 13 | 46.43% | 8 | 47.06% |
|  | **F** | 15 | 53.57% | 9 | 52.94% |
| **Age** | **Min** | 36 |  | 24 |  |
|  | **Max** | 81 |  | 58 |  |
|  | **Median** | 59 |  | 35.5 |  |
| **Ethnicity** | **Caucasian** | 25 | 89.29% | 1 | 5.88% |
|  | **Asian** | 1 | 3.57% | 3 | 17.65% |
|  | **Black/ African/ Caribbean** | 2 | 7.14% | 1 | 5.88% |
|  | **Other/ Mixed** | 0 | 0.00% | 1 | 5.88% |
|  | **UA** | 0 | 0.00% | 10 | 58.82% |
| **NEN Aetiology** | **Bowel NEN** | 15 | 53.57% |  |  |
|  | **Pancreatic NEN** | 11 | 39.29% |  |  |
|  | **NEN of Other Origin** | 2 | 7.14% |  |  |
| **Grade** | **G1** | 7 | 25.00% |  |  |
|  | **G2** | 11 | 39.29% |  |  |
|  | **G3** | 2 | 7.14% |  |  |
|  | **UA** | 8 | 28.57% |  |  |

**Table 1S.** Clinical characteristics of the cohorts of LM-NEN patients and healthy controls (HC) utilised to quantify plasma levels of soluble immune checkpoints.
